# Supplementary material for: Co-Treatment of Erythroid Cells from β-Thalassemia Patients with CRISPR-Cas9-Based β039-Globin Gene Editing and Induction of Fetal Hemoglobin
Source: Genes (Basel). 2022 Sep 26;13(10):1727. doi: 10.3390/genes13101727 (PMC9601852; doi:10.3390/genes13101727)
Supplement: Supplementary file 1 [file genes-13-01727-s001.zip › genes-1846681-supplementary.pdf]

genes

MDPI

Co-treatment of erythroid cells from β-thalassemia patients with CRISPR-Cas9-based $\beta^0$39-globin gene editing and induction of fetal hemoglobin

Lucia Carmela Cosenza [1], Cristina Zuccato [1,2], Mattreo Zurlo [1], Roberto Gambari [2*] and Alessia Finotti [1,2,*]

[1] Department of Life Sciences and Biotechnology, Section of Biochemistry and Molecular Biology, Ferrara University, 44121 Ferrara, Italy

[2] Center 'Chiara Gemmo and Elio Zago' for the Research on Thalassemia, University of Ferrara, 44121 Ferrara, Italy

* Correspondence: alessia.finotti@unife.it (A.F.) Tel.: +390532-974510; gam@unife.it (R.G.) Tel.: 39-0532-974443

Supplementary Materials

Supplementary Methods

SM1: Erythroid precursor cells (ErPCs) isolation and ErPC culture

ErPCs cultures were prepared from 25 mL of peripheral blood, using tubes treated with Vacutainer LH (BD Vacutainers, Becton Dickinson, United Kingdom), collected from $\beta^0$39-thalassemia homozygous patients (transfusion-dependent) before undergoing the patient's routine blood transfusion [43,58,59]. The PBMCs, obtained after centrifugation of the whole blood sample by Ficoll-Hypaque density gradient (Lympholyte®-H Cell Separation Media, Cedarlane, Euroclone, Italy) were collected, washed with 1X DPBS W/O CA-MG (GIBCO, Invitrogen, Life Technologies Carlsbad, CA, USA) and immediately after were maintained in Fibach phase I culture medium [58,59] containing an α-minimum essential medium (α-MEM, Sigma-Genosys, Saint Louis, Missouri, USA), prepared from a powder and diluted with water; 10% FBS (Celbio, Milan, Italy), a PEN-STREP solution (PEN-STREP 10000 U/mL, Lonza, Verviers, Belgium), 10 % conditioned medium (CM), obtained from cell cultures of bladder cancer cells 5637, 1 μg/mL of cyclosporin A (Sigma-Aldrich) prepared with cyclosporine absolute ethanol and diluted in 1X DPBS (GIBCO) in the ratio 1:1 and stem cell factor (SCF, Life Technologie, Carlsbad, California, USA) at a final concentration of 10 ng/mL. After 7 days in this culture medium, the non-adherent cells were collected, washed once with 1X DPBS (GIBCO), then grown in Fibach phase II medium [58,59] consisting of α-MEM (Sigma Genosys), 1% deionized bovine serum albumin (BSA, Sigma Genosys), 30% FBS (Celbio), L-glutamine 2 mM (Sigma Genosys), Dexamethasone 10^{-6} M (Sigma Genosys), 10^{-5} M β-mercaptoethanol (Sigma Genosys), stem cell factor (SCF, Life Technologie, Carlsbad, California, USA) at 10 ng/mL final concentration and 1 U/mL of recombinant human erythropoietin (EPO Tebu-bio, Magenta, Milan, Italy). The yield in terms of percentage of ErPCs was always higher than 85%, as suggested by immunological flow-cytometry (FCS) characterization using antibodies recognizing CD71 and CD235a, in agreement with previously reported data [58]. Representative FCS data and morphological analysis are shown in Figures S3 and S4. Elsewhere published data demonstrate that the large majority of ErPCs undergo erythroid differentiation, as demonstrated by FCS analysis using antibodies against transferrin receptor and glycophorin [60]. We carefully considered the fact that FBS might heavily affect ex vivo erythroid differentiation and hemoglobin production, thereby creating variability. For this reason, we screened all the FBS batches, selecting only those lacking effects on differentiation of ErPCs and on HBF production after exposure to HbF inducers. Moreover, the same batch of FBS was always used throughout all the experiments reported in the present study.

SM2: Flow Cytometry-based characterization of in vitro cultured ErPCs.

To verify the expression of typical markers of erythropoiesis (CD71 / d235a) by flow cytometry, 1 million cells in culture with erythropoietin for two weeks were collected. The cells were washed in PBS and subsequently labeled with 10 ul of antibody against CD71 (Miltenyi Biotec CD71-FITC cat.n.130-098-779) and 10 ul of antibody against CD235a (Miltenyi Biotec Glycophorin A-PE cat.n.130-100-259). After 15 min of incubation in the dark, cells are washed twice in PBS and resuspended in 200 μl of PBS before acquisition. Samples were acquired using a BD FACSCanto™ II Flow-Cytometry system and obtained data analyzed using FlowJo v.10 software.

SM3: Cytospin and morphological analysis of ErPCs.

$1x10^5$ cells are concentrated in 100 ul of PBS and subsequently loaded through a special reservoir on a microscope slide by cytocentrifuge (5 min at 550 rpm), prepared slides are dried overnight under a chemical hood. The next day the glasses are covered with undiluted May Grunwald dye for 4 minutes (ethanol contained in this solution is sufficient to fix cells on the slide), then an equal volume of double distilled water is added to each glass and incubated another 4 minutes. At the end of the incubation, the glasses are drained and then covered with GIEMSA dye diluted

1:10 and incubated for another 12 minutes. Finally, the glasses are washed with abundant double-distilled water and dried overnight under a chemical hood. The prepared slides were then analyzed with a Nikon eclipse 80i optical microscope using a 60x oil immersion objective and captured with Nikon NIS Element imaging software.

SM4: Cell electroporation with CRISPR-Cas9 system for correction of the $\beta^0$39-globin gene mutation

The genomic sgRNA target sequence was 5′-<u>TGGTCTACCCTTGGACCTAG</u>**AGG**-3′ (sgRNA target sequence underlined, PAM in bold); the gRNA complex begins by joining a tracrRNA (ATTO 550 labeled Alt-R® CRISPR-Cas9 tracrRNA, IDT), and the Alt-R® CRISPR-Cas9 crRNA (IDT) oligonucleotide in thermoblock at 95° C for 5 minutes. Then, Cas9 RNP was made by incubating gRNA and Cas9 at a molar ratio of 1:2 at 25°C for 10 min immediately before electroporation. After 3 days of cell culture, according to the phase II of Fibach's protocol [43,59], the ErPCs have been electroporated in the presence of 6 μM concentration of β-globin PAM-modified donor template with nucleotides chemically modified at both the 5′ and 3′ ends (the nucleotides contained 2-O-methyl-3′-phosphorothioate moieties (IDT, Tema, Coralville, Iowa, USA)); Cas9 RNP was also included [24]. All components were resuspended with cells and electroporated in Mirus Ingenio® solution (Mirus Bio LLC, Madison, USA), in a final volume of 100 μL, using the Lonza Nucleofector IIb (program U-008). After the electroporation, the cells were grown in the Fibach phase II culture medium as described above or, for the combined approach experiment, in the Fibach phase II culture medium with the addition of 200 nM rapamycin. After 5 days the erythroid precursors were collected to carry out the various analyses on DNA, RNA, and proteins.

SM5: Genomic DNA Extraction

The DNA was extracted from 200-300 mL of whole blood using QiAmp DNA Blood Mini Kit (QIAGEN, Hilden, Germany) according to the manufacturer's protocol. The DNA obtained was visualized on a UV transilluminator after 0.8 % agarose gel electrophoresis and quantified using the spectrophotometer SmartSpec™ Plus (Biorad Smartspec Plus, Bio-Rad).

SM6: Droplet Digital PCR (ddPCR) to evaluate genomic and transcriptomic $\beta^0$39 globin correction

The evaluation of the β-globin gene correction levels in the position of codon 39 was carried out by Droplet Digital PCR [24,62]. In these experiments, Taq-Man probes marked with FAM and HEX fluorophores were used, designed specifically for the identification of the sequence containing the $\beta^0$39 mutation (HEX) in the β-globin gene and the corresponding corrected sequence (FAM) (Table 1). Both probes were used for genomic and transcriptional analysis and were designed using IDT tools. While specific pairs of primers have been designed for the selective amplification of DNA or transcript (in introns in the case of genomic analysis, and in exons in the case of transcriptomic analysis). The predetermined quantity of DNA or cDNA obtained following extraction from erythroid precursor cells treated with the CRISPR-Cas9 system was added to ddPCR reaction mix containing 2X ddPCR Supermix for Probes (no dUTP) (Bio-Rad) and 20X TaqMan $\beta^0$39 Assay or β edited Assay (IDT). The ddPCR reaction was mixed with Automated Droplet Generation Oil for Probes (Bio-Rad) and droplets emulsion (water in oil) was automatically generated using Automated Droplet Generator (AutoDG) (Bio-Rad). The emulsion was amplified using GeneAmp PCR System 9700 (Thermo Fisher Scientific) using the following thermal cycler condition at 95° C for 10 minutes, 94° C for 30 seconds and 61° C for 1 minute repeated for 40 cycles, then a final phase of inactivation of the enzyme DNA polymerase at 98° C for 10 minutes. The plate must be kept at 4° C for one hour before reading to stabilize the analysis. Generated droplets were read using the QX200 Droplet Reader, and data analysis was performed with QuantaSoft version 1.7.4 (Bio-Rad).

SM7: HPLC Analysis of Hemoglobins

In order to evaluate the correction of the β-globin gene following treatment with the genome editing system in erythroid precursor cells, determining the increase in the level of HbA produced, an HPLC analysis was performed on the protein extracts. The ErPCs were centrifuged at 8000 rpm for 8 minutes and washed with PBS (Phosphate buffered saline). Cells, pelleted and resuspended in HPLC-grade water (Sigma-Aldrich, St. Louis, Missouri, USA), undergo 3 freeze/thaw cycles on dry ice to lyse the cells and obtain the protein extracts. Hemoglobin analysis is performed by loading the protein extracts into a PolyCAT-A cation exchange column and then eluted in a sodium-chlorine-BisTris-KCN aqueous mobile phase using HPLC Beckman Coulter instrument System Gold 126 Solvent Module-166 Detector which allows to obtain a quantification of the hemoglobins present in the sample. The reading is performed at a wavelength of 415 nm, and a commercial solution of purified human HbAF (Sigma-Aldrich) extracts has been used as standard. The values thus obtained are processed using "32 Karat software" [61,63].

SM8: Western Blotting Analysis

The accumulation of the β-globin protein (16 kDa) and the increase in γ-globin protein (15 kDa) in the presence of induction with rapamycin were assessed by Western blotting. For whole-cell extract preparation, the cells were lysed through three freeze-thaw cycles in dry ice and quantified by BCA assay (PierceTM BCA Protein Assay kit, Thermo Fisher Scientific). For each sample 20 μg of ErPCs extracts were loaded on 16% acrylamide SDS-PAGE gel (40% Acrylamide/bis solution, BioRad). After separation by an electrophoretic run, the proteins were transferred onto nitrocellulose paper, and incubated with different primary antibodies: anti-β-globin chain antibody (mAb, sc-21757, Santa Cruz), anti-γ-globin chain antibody (pAb, PA5-29006, Thermo) and a constitutive protein anti-GAPDH (mAb, MA1-16783, Thermo) used as housekeeping to normalize the quantification of the target protein. The quantification of the bands was carried out by ChemiDoc (Bio-Rad) densitometric analysis.

SM9: HPLC-based analysis of the excess of "free α-globin chains"

Lysates from $2x10^5$ ErPCs (either untreated or GE and GE + RAPA treated) were prepared and 100 μl were analyzed by HPLC as described in the Materials and Methods section of the main manuscript. Beckman Coulter instrument System Gold 126 Solvent Module-166 Detector was used, which allows obtaining a quantification of the peaks present in the sample. The reading was performed at a wavelength of 415 nm, and a commercial solution of purified human HbAF (Sigma-Aldrich) extracts has been used as standard. The values thus obtained are processed using "32 Karat software" [61,63]. For further details on the HPLC characterization of hemoglobins and α-globin chain peak, see Zuccato et al. [58].

Supplementary Figures

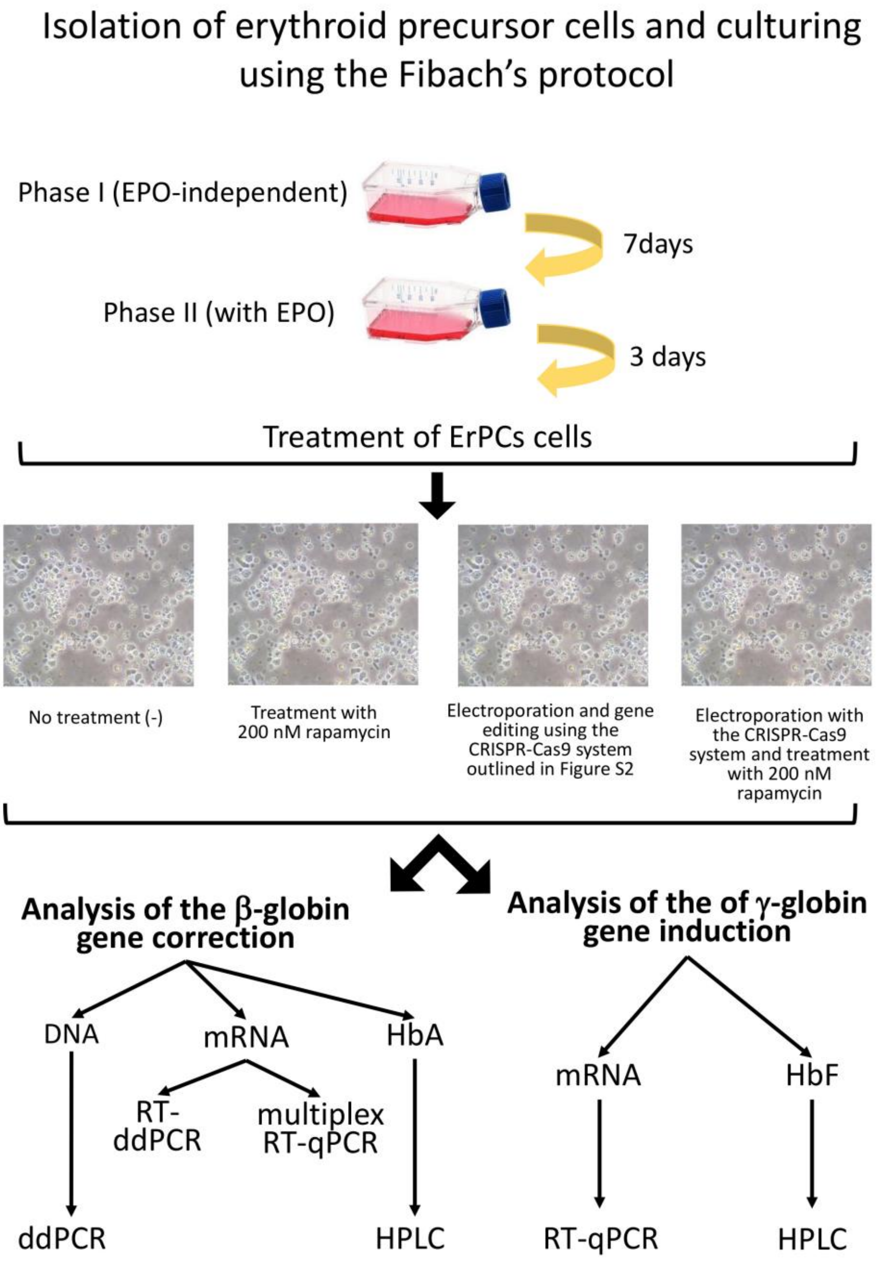

Figure S1. Scheme of the experimental strategy used for the correction of the $\beta^0$39-thalassemia mutation and the simultaneous induction of fetal hemoglobin in ErPCs isolated from homozygous $\beta^0$39 patients. The isolated cells are cultured using the Fibach method and on the third day of phase II are treated with rapamycin and electroporated with the $\beta^0$39 CRISPR-Cas9 system and the combined inductor and $\beta^0$39 CRISPR-Cas9 system, respectively. After the treatment, the cells were kept in culture for a further 5 days and then analyzed to evaluate the correction of the $\beta^0$39-thalassemia mutation at the DNA, mRNA and protein levels for β-globin and mRNA and protein levels for γ-globin.

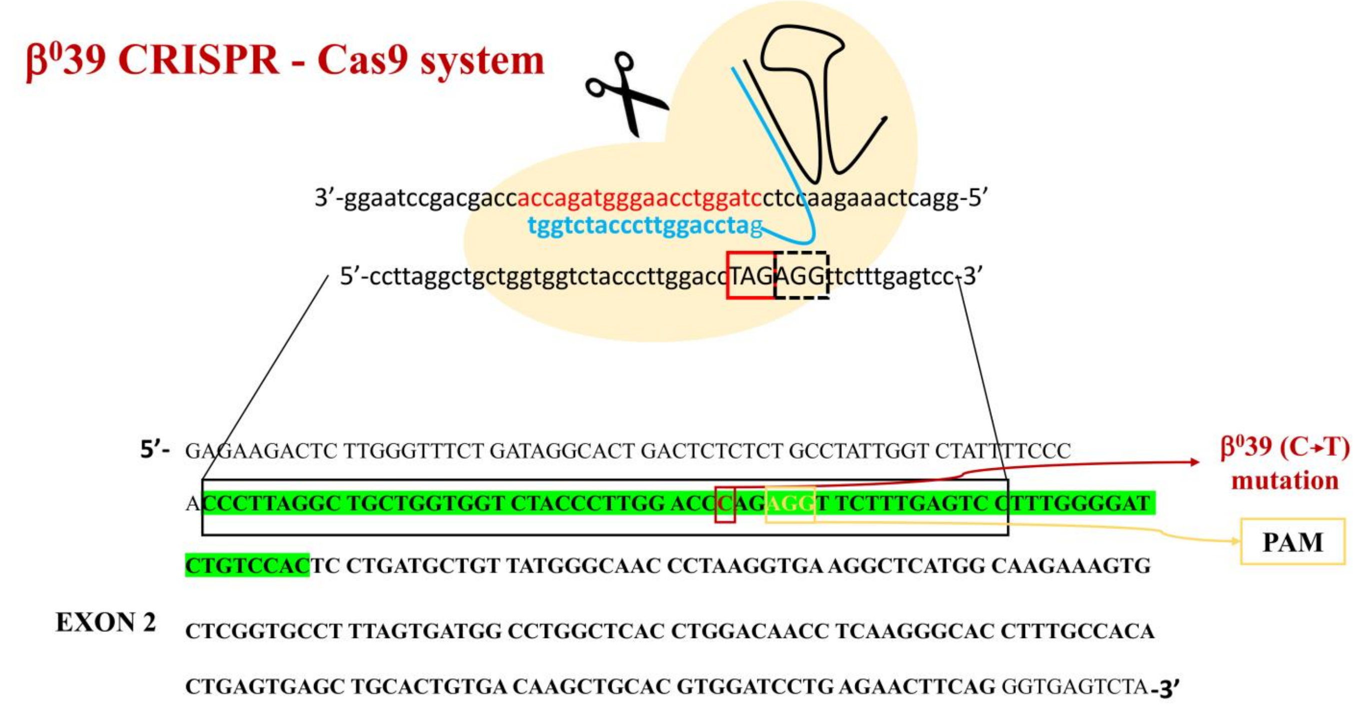

Figure S2. Model of the β039 CRISPR-Cas9 system used for gene editing experiments. Representation of the CRISPR-Cas9 system that recognizes and cuts DNA at exon 2 of the β-globin gene. The nucleotide sequence used as a template donor for the correction of the β039 thalassemia mutation is highlighted in green. The β039 (C→T) mutation and the PAM sequence are indicated in red and yellow, respectively.

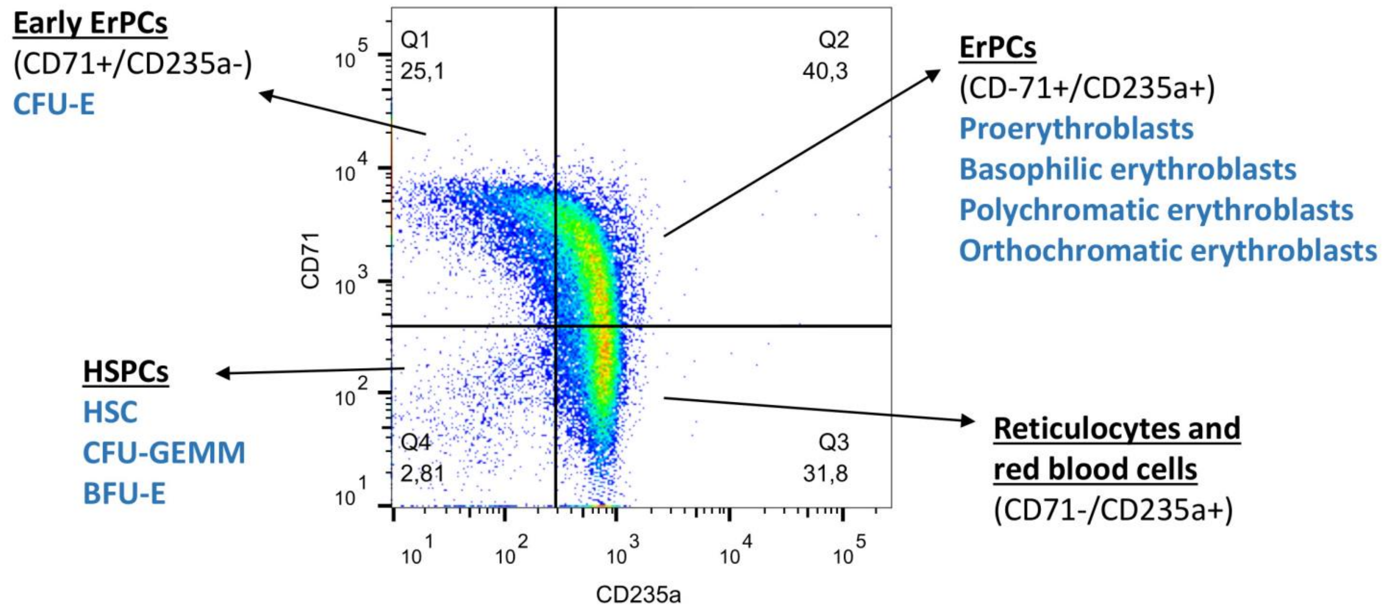

Figure S3. FCS analysis of ErPCs. Representative flow-cytometry analysis of ErPCs cultivated for a week in Fibach phase II culture medium. Most of the cells are positive for both erythroid markers CD71 (Transferrin receptor) and CD235a (Glycophorin A) while others are positive only for CD71 or CD235a, demonstrating the concomitant presence of different stages of erythroid differentiation and good overall purity of the cell culture.

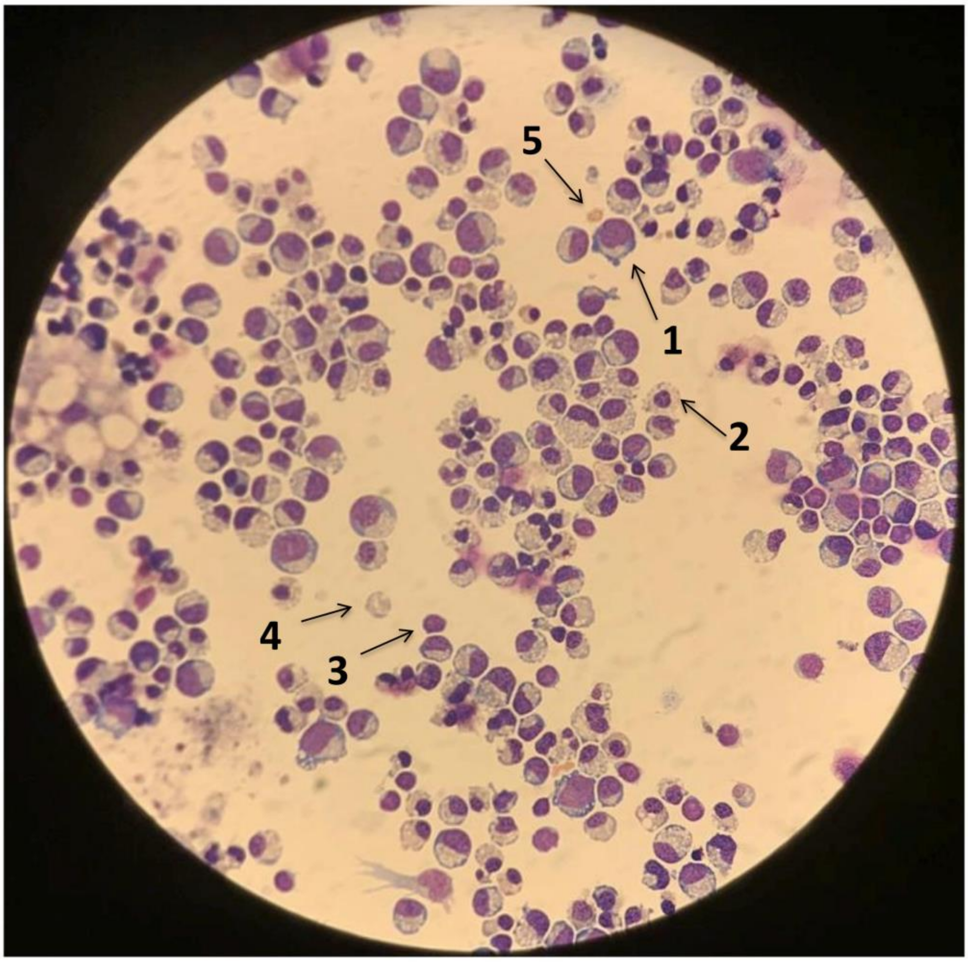

Figure S4. **Cytospin analysis from cultured ErPCs from a $\beta^0$39-thalassemia patient**. Representative cytospin image of ErPCs cultivated for a week in Fibach phase II culture medium and stained with May Grünwald Giemsa, the majority of the cells visualized are in the medium-late stages of erythroid differentiation, orthochromatic erythroblasts are the most prevalent population but also some reticulocytes and mature red blood cells are present. 1: Proerythroblast; 2: Orthochromatic erythroblasts; 3: Pyrenocytes; 4: Reticulocytes; 5: Mature red blood cells.

Amplicon results on $\beta^0 39$ target site

SAMPLE NAME	REPLICATE ID	TOT READS	READS "T" ALLELE	**%AF "T"**	READS "C" ALLELE	**%AF "C"**	READS DEL	**%AF DEL**	READS INS	**%AF INS**
Th-C-	1	461288	459674	**99.7**	893	**0.2**	23	**0**	0	**0**
	1A	262450	261364	**99.6**	557	**0.2**	53	**0.02**	0	**0**
Th-GE	2	377829	31396	**8.3**	26798	**7.1**	35671	**9.4**	538	**0.1**
	2A	262572	218433	**83.2**	23086	**8.8**	19472	**7.4**	1206	**0.5**

Figure S5. Editing results obtained by amplicon sequencing from ErPCs isolated from a $\beta^0 39$ thalassemia patient. The representative data reported in the table show the sample name (C-): untreated cells and GE (cells treated with the CRISPR-Cas9 system), the total number of reads containing the base position (chr11:5,226,774, corresponding to the $\beta^0 39$ mutation), the number of reads containing the allele T (mutated); the allele frequency of T, the number of reads containing the allele C (not mutated); the allele frequency of C, the number of reads containing the deletion; the allele frequency of deletion, the number of reads containing the insertion; the allele frequency of insertion.

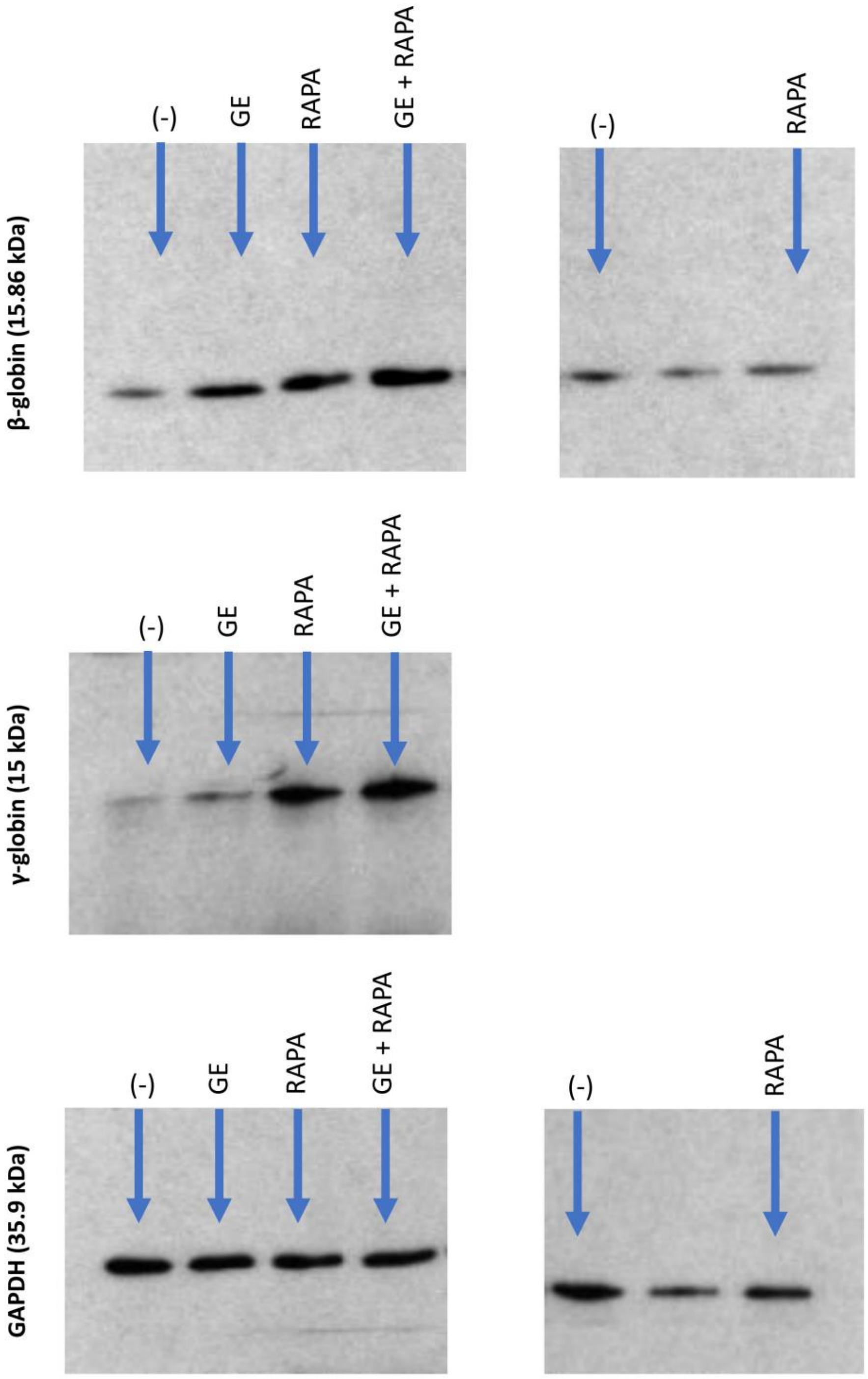

Figure S6. Uncut version of the Western blots: Protein analysis by Western blotting on ErPCs cultures treated with the combined system of CRISPR-Cas9 and rapamycin. The gels report the results of Western blotting analyses conducted on different cultures of ErPCs isolated from $\beta^0 39$ thalassemia patients. The cellular lysates were analyzed in order to investigate the presence of the proteins β-globin and γ-globin respectively and by interspersing very strong membrane stripping steps in order to avoid non-specific binding of the different antibodies used. The data obtained were normalized using the GAPDH protein as a reference. The bands shown in the figure are C(-): untreated cells; GE (cells treated with the CRISPR-Cas9 system), RAPA (rapamycin 200 nM) and GE + RAPA (cells treated with the CRISPR-Cas9 system and then cultured in the presence of rapamycin).

A

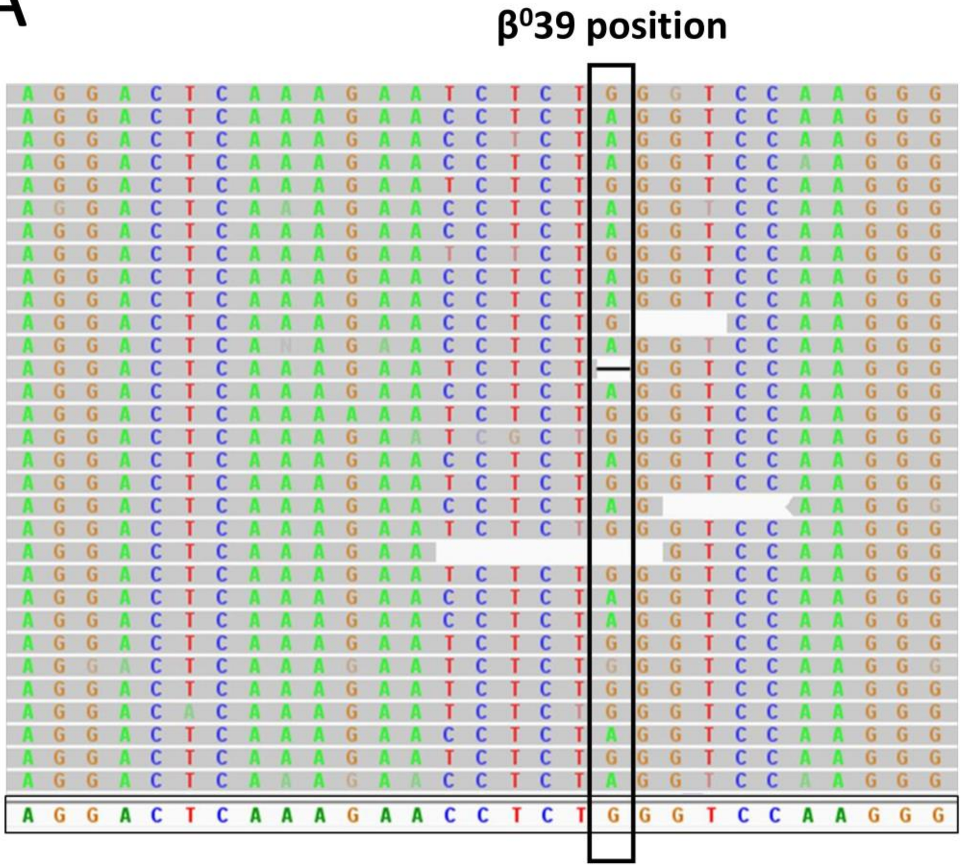

B

Editing WGS results on $\beta^0$39 target site

	CHR	POS	TOT # OF READS	READS "T" ALLELE	%AF "T"	READS "C" ALLELE	%AF "C"	READS "DEL"	%AF DEL	READS "INS"	%AF INS
Th-C-	CHR11	5226774	43	43	**100%**	0	**0%**	0	**0%**	0	**0%**
Th-GE	CHR11	5226774	32	24	**75%**	4	**13%**	3	**9%**	0	**0%**

Figure S7. Alignment of the fragments generated by the WGS sequencing obtained from an ErPCs sample edited with the $\beta^0$39 CRISPR-Cas9 system. (A) The figure shows sections of some representative reads obtained following the WGS sequencing of the ErPCs isolated from a $\beta^0$39 thalassemia patient. In particular, the alignment relative to position chr11:5,226,774 (in which the $\beta^0$39 thalassemia mutation is present) is indicated. In addition to the four nucleotides reported each with a different color, deletions are visible, shown with a black line. The empty spaces indicate the adjacent portions of the reads alignment. The reads were aligned by placing the same unedited patient as the reference genome sequence reported in the nucleotide sequence below. (B) The table shows the sample name (C-): untreated cells and GE (cells treated with the CRISPR-Cas9 system), the total number of reads containing the base position (chr11:5,226,774, corresponding to the $\beta^0$39 mutation), the number of reads containing the allele T (mutated); the allele frequency of T, the number of reads containing the allele C (not mutated); the allele frequency of C, the number of reads containing the deletion; the allele frequency of deletion, the number of reads containing the insertion; the allele frequency of insertion.

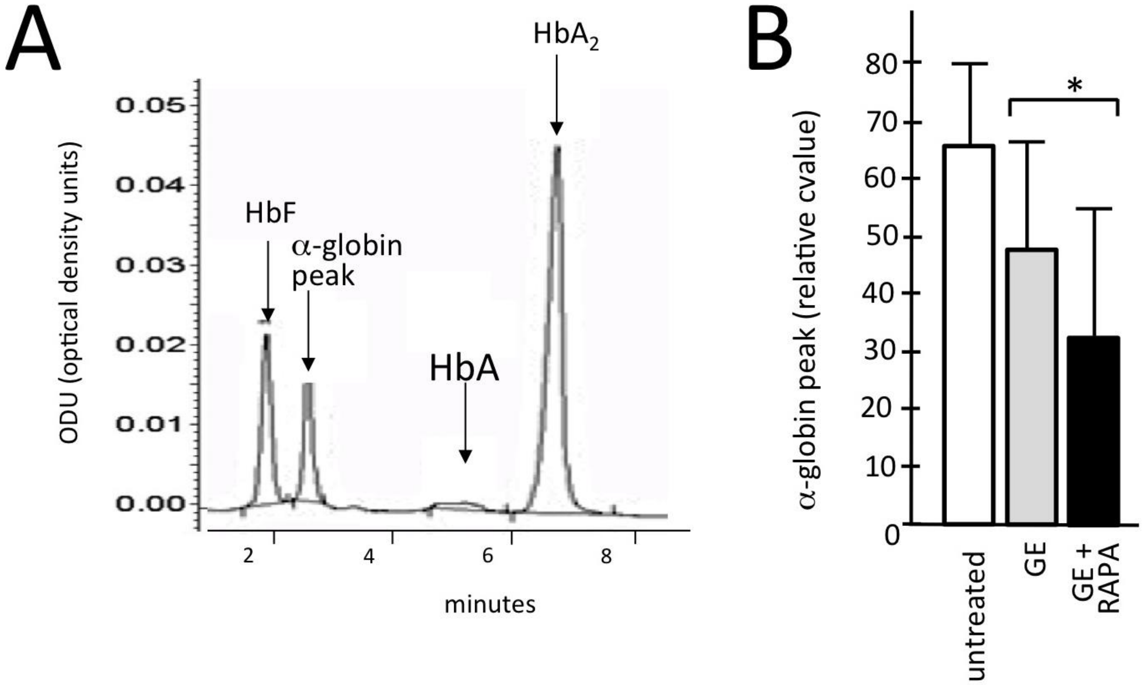

Figure S8. Reduction of the free α-globin peak after rapamycin treatment of gene-edited ErPCs. (A) Representative HPLC analysis of ErPC lysate from a $\beta^0$39-thalassemic patient recruited for this study. The α-globin chain peak is arrowed, together with the HbF, HbA and HBA2 peaks. For further details on the HPLC characterization of hemoglobins and α-globin chain peak, see and Zuccato et al. [58]. (B) Reduction of the free α-globin peak after rapamycin treatment of gene-edited ErPCs (GE + RAPA). The data are the average ± S.D. of four independent experiments. * = $p < 0.05$.
